# Morphogen-driven differentiation is precluded by physical confinement in human iPSCs spheroids

**DOI:** 10.3389/fbioe.2024.1467412

**Published:** 2024-11-11

**Authors:** Haneen S. Alsehli, Errin Roy, Thomas Williams, Alicja Kuziola, Yunzhe Guo, Cecile A. Dreiss, Jeremy B.A. Green, Eileen Gentleman, Davide Danovi

**Affiliations:** ^1^ Centre for Gene Therapy and Regenerative Medicine, King’s College London, London, United Kingdom; ^2^ Centre for Stem Cell Biology, University of Sheffield, Sheffield, United Kingdom; ^3^ Centre for Craniofacial and Regenerative Biology, King’s College London, London, United Kingdom; ^4^ Institute of Pharmaceutical Science, King’s College London, London, United Kingdom; ^5^ Department of Biomedical Sciences, University of Lausanne, Lausanne, Switzerland; ^6^ Department of Basic and Clinical Neuroscience, King’s College London, London, United Kingdom; ^7^ Migration Biotherapeutics, Cardiff, United Kingdom

**Keywords:** pluripotent stem cells, morphogenesis, germ layer differentiation, PEG-based hydrogels, high content image analysis

## Abstract

**Introduction:**

Cell lineage specification is tightly associated with profound morphological changes in the developing human embryo, particularly during gastrulation. The interplay between mechanical forces and biochemical signals is poorly understood.

**Methods:**

Here, we dissect the effects of biochemical cues and physical confinement on a 3D *in vitro* model based on spheroids formed from human induced pluripotent stem cells (hiPSCs).

**Results:**

First, we compare self-renewing versus differentiating media conditions in free-floating cultures and observe the emergence of tri-germ layers. In these unconfined conditions, BMP4 exposure induces polarised expression of SOX17 in conjunction with spheroid elongation. We then physically confine spheroids using PEG-peptide hydrogels and observe dramatically reduced SOX17 expression, albeit rescued if gels that soften over time are used instead.

**Discussion:**

Our study combines high-content imaging, synthetic hydrogels, and hiPSCs-derived models of early development to define the drivers that cause changes in the shape and the emergence of germ layers.

## Introduction

During the early stages of human development, the pluripotent stem cells of the epiblast undergo gastrulation, breaking symmetry to generate anterior-posterior (A-P) axial elongation and shaping the body plan. The morphogenesis process is temporally associated with lineage specification; the pluripotent stem cells eventually give rise to all cell types in the mammalian body (Moris et al., 2020). The interactions between these cells and the extra-embryonic environment are vital regulators of morphogenesis and gastrulation events in mammals (Muncie et al., 2020; Vianello and Lutolf, 2019). Prior to gastrulation, the blastocyst becomes embedded into the uterine wall, thus providing mechanical interaction between the cells and the extracellular matrix (ECM) (Vianello and Lutolf, 2019). Cells are subjected to different cues, including biochemical or mechanical signals. These include secreted morphogens, growth factors, and mechanical forces from the surrounding microenvironment (Muncie et al., 2020). These signals influence cellular behaviour, drive axial elongation and induce lineage differentiation to form the three germ layers. However, the interplay between mechanical interactions that drive morphogenesis and lineage commitment during early development is challenging to study *in vivo* and poorly understood (Vianello and Lutolf, 2019; Trubuil et al., 2021).

Human pluripotent stem cells (hPSCs), including human induced pluripotent stem cells (hiPSCs) and human embryonic stem cells (hESCs), offer an unprecedented tool to study cell fate decisions (Yu et al., 2021), self-organisation, and early developmental events (Yu et al., 2021; Rowe and Daley, 2019). hPSCs have been effectively used in 2D micropatterned systems to investigate the signalling pathways underlying self-organisation and fate decisions in early human development. Warmflash et al. demonstrated that hPSCs cultured on defined patterns could radially organise and differentiate into cells of the three germ layers following BMP4 treatment, mirroring early development (Moris et al., 2020; Warmflash et al., 2014; Tewary et al., 2017; Siggia and Warmflash, 2018). The molecular signals regulating self-organisation during the specification of the three germ layers have been investigated *in vitro* using various models (Vianello and Lutolf, 2019; Warmflash et al., 2014). Building on these findings, it has been proposed that mechanical tension generated by cell-adhesion in 2D gastrulation models is a crucial regulator of cell fate specification (Muncie et al., 2020; Vickers et al., 2021; Grolleman et al., 2023). Here, different geometrical shapes (circle, square, and triangle) alter BMP4-mediated patterning in regions of high tension (Muncie et al., 2020). These approaches have advanced our knowledge of the role of geometry in lineage commitment and signalling (Warmflash et al., 2014; Bauwens et al., 2008). In other organoid systems (Warmflash et al., 2014; Sato and Clevers, 2013), the dynamic interactions between the intrinsic mechanical properties of the surrounding tissue (Petzold and Gentleman, 2021) and the forces and soluble signals cells generate and receive affect cell behaviour (Muncie et al., 2020; Vianello and Lutolf, 2019).

On the other hand, 3D cultures offer a more realistic model to investigate the role of physical forces in response to environmental cues in conjunction with morphogen-triggered signalling events. Various culture methods are proposed to establish embryo-like axial elongation and display key features, including gene expression patterns that mirror normal development (Moris et al., 2020; Hashmi et al., 2021; Bedzhov and Zernicka-Goetz, 2014). A recent study described that hESCs cultured in suspension under defined conditions treated with pulses of the Wnt agonist (Chiron) drove symmetry breaking and elongated morphologies in the absence of extra-embryonic tissues (Moris et al., 2020; Baillie-Benson et al., 2020). The resulting “gastruloids” exhibited polarised expression of mesoderm (BRA), endoderm (SOX17) and neuroectoderm (SOX2) markers. These observations suggest that Wnt signalling is sufficient to initiate axial elongation and patterning of the three germ layers in 3D hiPSCs cultures. In contrast to Chiron pulse treatment, in the presence of BMP4 under similar conditions, cells were reported to fail to aggregate, elongate and pattern (Moris et al., 2020). Notably, different culture strategies have been proposed, such as exposing hPSC to mTesR medium, mouse embryonic fibroblast conditioned medium, or E6 in the presence of BMP4 or Chiron to induce lineage specification. Overall, both 2D and 3D culture systems demonstrate the ability of morphogens such as BMP4 and Wnt agonists to induce germ layers formation (Warmflash et al., 2014; Tewary et al., 2017; Vickers et al., 2021).

In addition, the physical properties of the tissue microenvironment are also crucial before and during lineage specification *in vitro* to achieve patterning and axial elongation (Moris et al., 2020). Hydrogels can be used to manipulate mechanical properties in 3D *in vitro* models of gastrulation (Vianello and Lutolf, 2019; Grolleman et al., 2023; Blache et al., 2022). Such platforms can control matrix stiffness, degradability and cell adhesion and have been used to study the role intrinsic mechanical cues play in supporting intestinal organoids and controlling neural tube morphogenesis (Ranga et al., 2016; Gjorevski et al., 2016). Hydrogels provide well-defined, reproducible environments that control both physical and biochemical cues (Gjorevski and Lutolf, 2017). Indeed, hydrogel stiffness can be tuned by varying the polymer concentration, and biological cues can be controlled by incorporating integrin-binding (RGD) and matrix metalloproteinase-degradable peptide sequences (Gjorevski et al., 2016). Matrix stiffness is a key regulator of multiple cellular processes, including proliferation, differentiation, migration and spreading. Notably, previous work showed intestinal organoid formation is favoured in hydrolytically degradable (Jowett et al., 2021) or viscoelastic (Chrisnandy et al., 2022) rather than purely elastic matrices. In hiPSCs, adhesion signalling directs morphogenic events such as lumen formation and apicobasal polarity in 3D hiPSCs (Indana et al., 2021; Seitz et al., 2024).

In the early stages of gastrulation, as the primitive streak is formed, the cells ingress inward and mesodermal cells (BRA^+^) are specified, inducing the emergence of endodermal progenitors (SOX17^+^) (Taha et al., 2016; Niakan et al., 2012; Niakan et al., 2010). Nevertheless, further investigation is needed to understand how these processes are coordinated to influence cell fate decisions. Indeed, the requirement for morphogenesis (if any) to achieve proper differentiation has not been clearly established (Fulton et al., 2020; Lecuit and Lenne, 2007). Here, we aimed to investigate how biochemical cues and physical confinement separately influence morphogenesis and differentiation in a 3D hiPSCs model. Observing and quantifying how spheroid-forming and cells behave within defined medium conditions upon BMP4 treatment. To specifically question whether morphogenesis is required to direct lineage differentiation and patterning (Chen et al., 2011; Tewary et al., 2019), we hypothesised that BMP4 provides sufficient signals to trigger symmetry breaking, elongation and differentiation of hiPSCs spheroids in suspension culture. We also hypothesised that synthetic hydrogels (Jowett et al., 2021; Lust et al., 2021) inhibit elongation, thus enabling us to interrogate whether physical confinement affects not just morphogenesis but also cell fate specification. Taking advantage of previous 2D micropattern systems (Warmflash et al., 2014; Minn et al., 2020), we adapted a similar set-up in our 3D model. When hiPSCs spheroids were encapsulated within hydrogels, elongation was impeded and SOX17 expression was dramatically reduced. Our approach enables us to investigate the inter-correlation of changes in shape with patterning of germ layers in hiPSCs-based models of early development.

## Results

### BMP4 signalling induces axial elongation in 3D hiPSCs spheroid models

**FIGURE 1:**
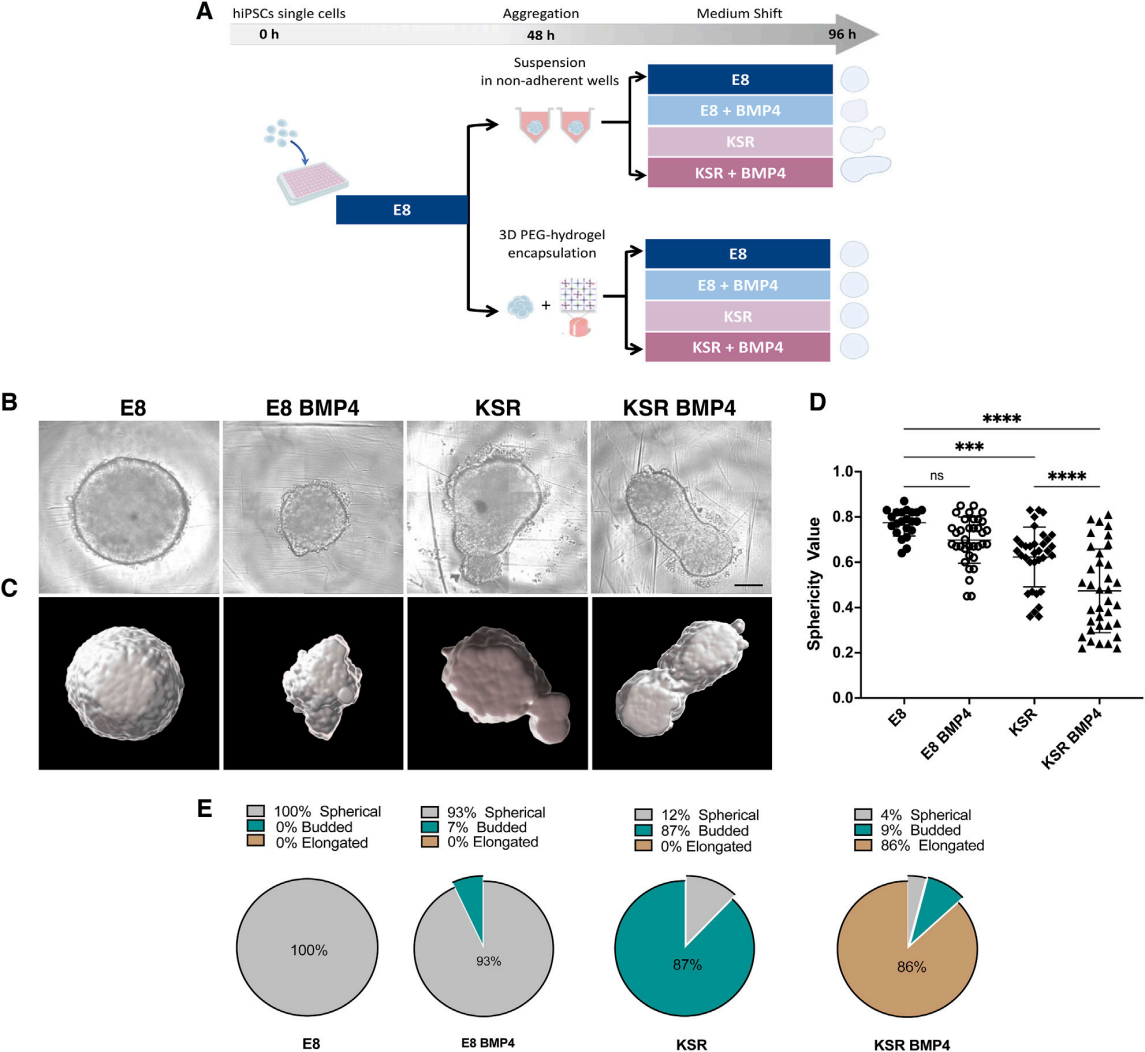
hiPSCs spheroids undergo distinct morphological change under different medium conditions. **(A)** Schematic of the hiPSCs spheroid formation protocol: hiPSCs single cells are seeded in low attachment well plates and cultured in E8 medium for 2 days to form spheroids. (top) free-floating spheroids in suspension; (bottom) spheroids encapsulated in PEG hydrogels. After 48 h, the medium is changed and cultured in E8 or KSR BMP4 or E8 BMP4 medium or KSR alone conditions. **(B)** Bright-field images exhibit changes in spheroid morphology post-96 h in culture. Scale bar 200 um. **(C)** Cell masked images created using Imaris software highlight deformation in shape enabling its quantification. **(D)** Analysis of shape deformation: sphericity values were obtained from the segmented images (n = 22, 36 individual spheroids in E8, and rest of conditions respectively, N = 10 experiments, ****P< 0.0001, ***P< 0.001, bars = mean and SD). Statistic test: ordinary one-way ANOVA, Šídák’s multiple comparisons test. **(E)** Quantification of the percentage of spheroids adopting spherical, budded and elongated morphologies in each medium condition (n = 230 individual spheroids, N = 10 experiments).

As BMP4 treatment has been shown to generate the three germ layers in 2D models, we developed a method to explore whether BMP4 triggers changes in shape of a 3D hiPSCs model. hiPSCs single cells were cultured under defined medium conditions that provided consistent shape variation in spheroid morphology (Figure 1A). E8 medium promotes self-renewing conditions, while KSR BMP4 triggers differentiation (Chen et al., 2011; Tewary et al., 2019). We then monitored spheroid formation of hiPSCs aggregates (Figure 1B; Supplementary Image S1).

To quantify dynamic changes in the morphology of hiPSCs spheroids in suspension, we first set up a robust high-content imaging-based platform to assess morphology using frame-to-frame variation analysis to obtain efficient segmentation of simple phase-contrast images and precise morphology quantification (i.e., size and shape) (Alsehli et al., 2021). Consistent with our previous results, we observed distinct phenotypic variations in each medium condition (Figures 1B–E). Cells cultured in E8 medium formed round spheres (Figures 1B, D, E; Supplementary Video S1), whereas in KSR BMP4, spheroids broke symmetry and exhibited axial elongation (Figures 1B, D, E; Supplementary Video S2). The control conditions produced intermediate phenotypes: in E8 BMP4, spheres were smaller in size, and tended to form dynamic small protrusions (Figures 1B, D–E; Supplementary Video S3) while culturing in KSR alone produced a budded morphology (Figures 1B, D, E; Supplementary Video S4). These results demonstrate that adding BMP4 to KSR medium enabled consistent morphological changes, including axial elongation.

### BMP4 treatment drives morphogenesis of hiPSCs 3D spheroids and induces tri-lineage specification

**FIGURE 2:**
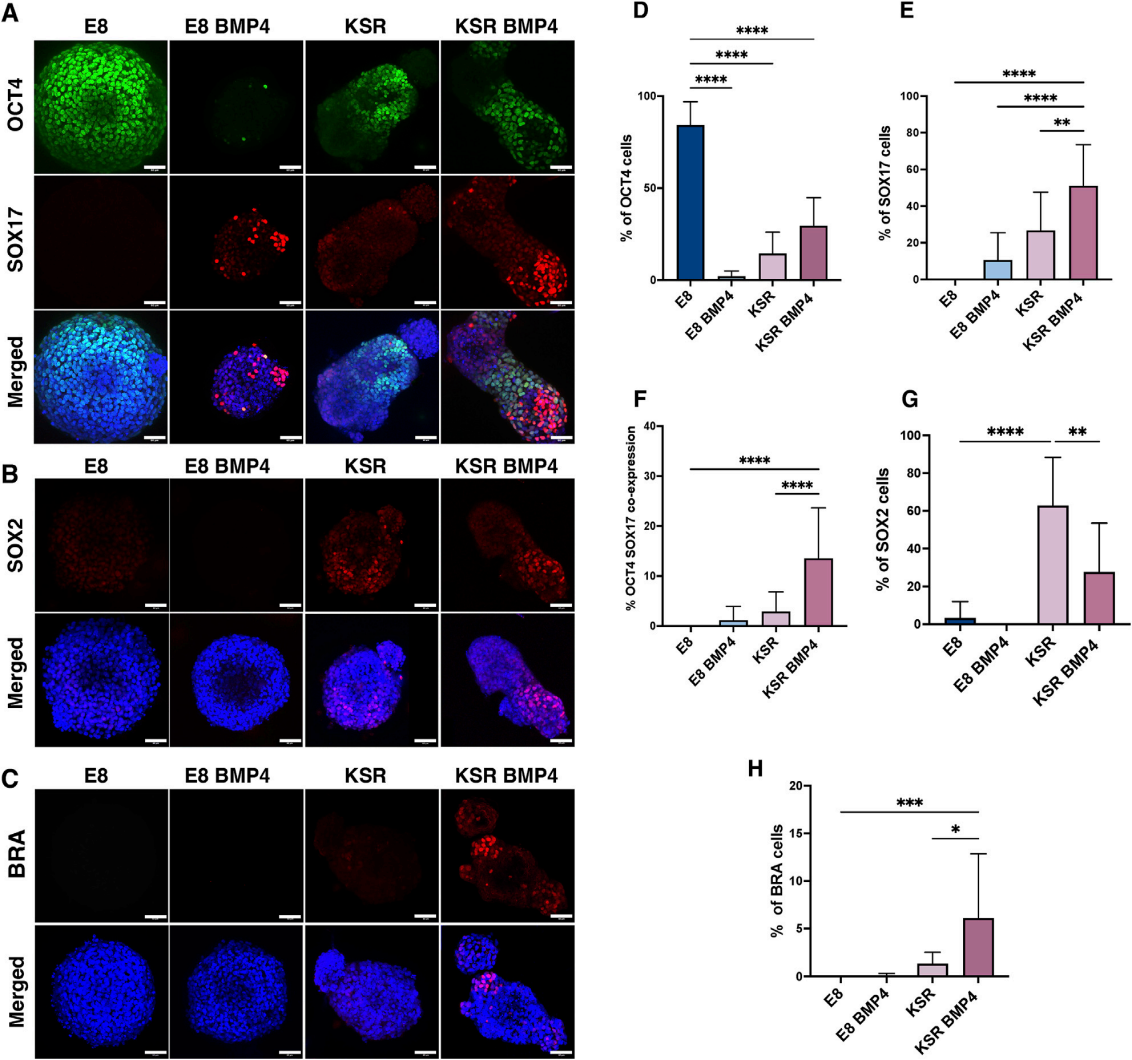
Emergence of tri-lineage specification in 3D hiPSCs spheroids in suspension. **(A–C)** Representative confocal microscopy images after 96 h of culture; spheroids in suspension are immunofluorescently labelled with lineage specification markers. OCT4 (pluripotency) and SOX17 (endoderm); SOX2 (ectoderm); BRA (mesoderm). Scale bars 60 µm. **(D)** Quantification of the percentage of cells expressing OCT4 per medium condition (n = 17 individual spheroids, N = 4 experiments, ****P< 0.0001, bars = mean value and SD). Statistic test: ordinary one-way ANOVA, Šídák’s multiple comparisons test. **(E)** Quantification of the percentage of cells expressing SOX17 in the different conditions (n = 13 individual spheroids, N = 4 experiments, ****P< 0.0001, **P< 0.005, bars = mean and SD). Statistic test: ordinary one-way ANOVA, Šídák’s multiple comparisons test. **(F)** Quantification of the percentage of cells co-expressing OCT4 and SOX17 markers (n = 16, N = 4 experiments, ****P< 0.0001, bars = mean and SD). Statistic test: ordinary one-way ANOVA, Šídák’s multiple comparisons test. **(G)** Quantification of the percentage of cells expressing SOX2 (n = 13 individual spheroids, N = 4 experiment, ****P< 0.0001, **P< 0.05, bars = mean and SD). **(H)** Quantification of the percentage of cells expressing BRA (n = 13 individual spheroids, N = 4 experiments, ***P< 0.001, *p = 0.01,bars = mean and SD). Statistic test: ordinary one-way ANOVA, Šídák’s multiple comparisons test.

To confirm differentiation concomitant with axial elongation, 3D hiPSCs were first stained for pluripotency marker OCT4 after 96 h of culture. This revealed that spheroids cultured in E8 medium expressed high levels of OCT4, while in the other three conditions, OCT4 was detected at low levels (Figure 2A).

We next interrogated markers for trilineage differentiation of germ layers as OCT4 expression is reduced. After 96 h, we stained for endodermal marker SOX17, mesodermal marker BRA, and ectodermal marker SOX2 to verify that 3D hiPSCs were able to form all lineages when cultured in KSR BMP4 media. Our results showed consistent emergence of the three lineage markers’ expression upon culture in KSR BMP4 compared to KSR medium (Figures 2A–H), which is known to only induce spontaneous differentiation (Taha et al., 2016; Ungrin et al., 2008).

Immunofluorescence staining showed that SOX17 was absent in spheroids grown in E8; however, strong expression of SOX17 in KSR BMP4 conditions was detected consistently in the distal domain of elongated spheroids in a mutually exclusive expression pattern with OCT4 (Figure 2A). Notably, in the KSR BMP4 medium condition, despite its ability to drive the most substantial shape change, we observed residual OCT4 expression, as well as another cell population co-expressing SOX17^+^/OCT4^+^ neighbouring SOX17^+^/OCT4^−^ cells in the elongated tip (Figure 2A). Consistent with this, when spheroids were cultured in E8 BMP4, low SOX17 expression was observed randomly. Morphological changes as previously described in (Figure 1B), demonstrated that a few spheroids in the E8 BMP4 medium condition formed small protrusions in which SOX17 was detected at low levels with marked polarisation in the protrusion area (Figure 2A). When spheroids were cultured in KSR medium, we observed lower expression of SOX17 (Figure 2A) that distributed across the entire spheroid compared to the KSR BMP4 condition (Figure 2A). SOX2, an ectoderm marker, and BRA, a marker for mesoderm, were expressed in KSR and KSR BMP4 conditions, but their expression patterns did not appear to correlate strongly with elongation (Figures 2B, C). Altogether, these observations indicate an associated lineage specification suggesting that morphogenesis and SOX17 polarisation toward the elongated area in KSR BMP4 could be coupled.

Next, we quantified SOX17 expression in 3D using Imaris software to estimate the percentage of cells expressing markers (Figures 2D–H). Unsurprisingly, hiPSCs spheroids cultured in E8 medium maintained high expression of OCT4 (Figure 2D). Conversely, a significant reduction of OCT4 expression was detected in all other medium conditions (E8 BMP4, KSR, and KSR BMP4), indicating that cells ceased to be pluripotent. Spheroids in KSR BMP4 medium had high levels of SOX17 positive cells compared to other conditions (Figure 2E). Quantification of ectoderm and mesoderm markers showed that spheroids cultured in E8 medium express low levels of the ectodermal marker SOX2 with no sign of BRA expression (Figures 2G, H). In the differentiating condition (KSR BMP4), SOX2 and BRA expression were observed (Figures 2G, H). When spheroids were grown in E8 BMP4 medium, neither SOX2 nor BRA was detected. Altogether, these observations demonstrate that in KSR BMP4 medium, spheroids give rise to cells that express markers for all three germ layers. In contrast, in KSR alone, spheroids undergo spontaneous differentiation primarily toward the ectodermal lineage.

### Biochemical cues drive changes in cell proliferation and cellular tension

**FIGURE 3:**
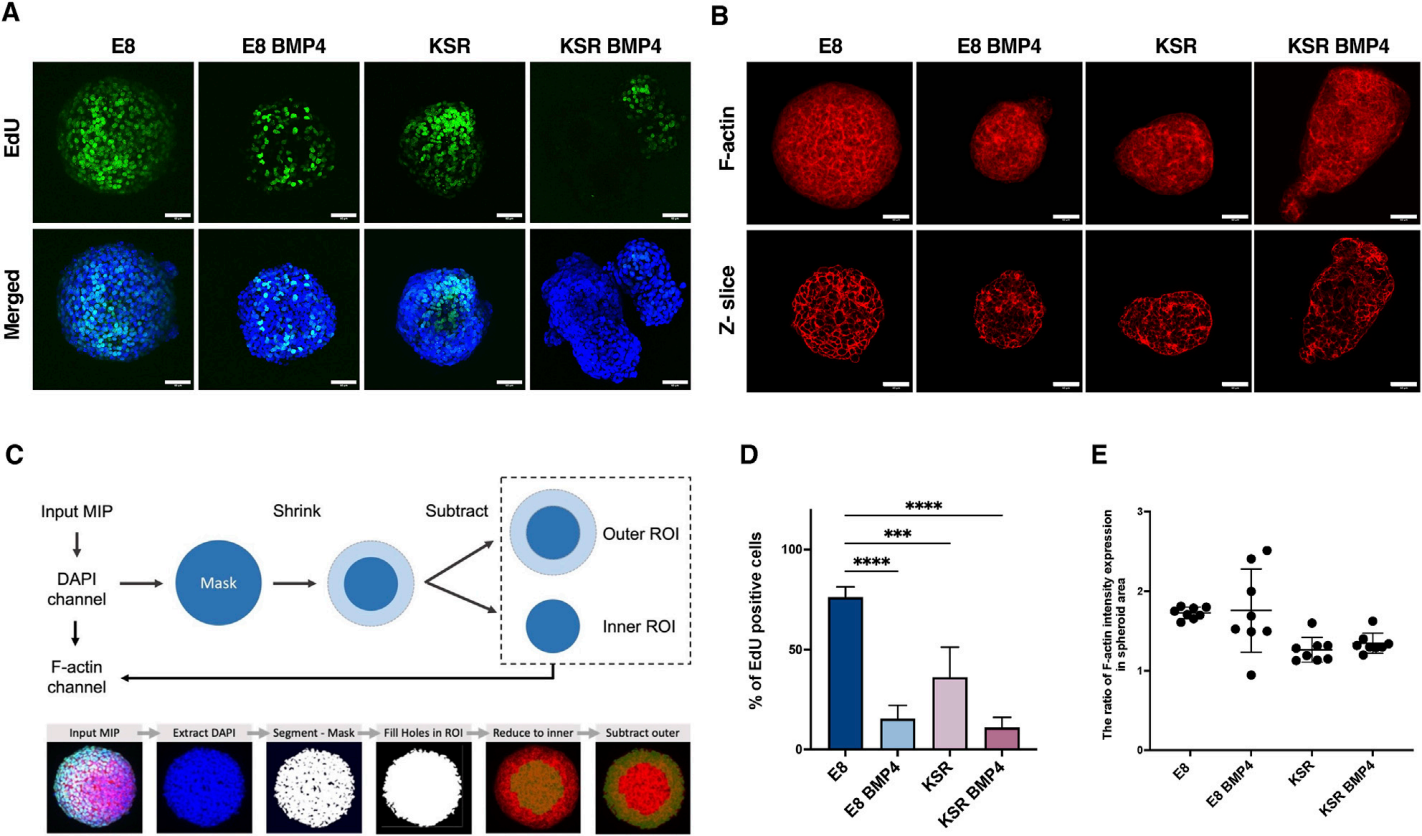
Comparison of cell proliferation and F-actin distribution across distinct culture conditions. **(A)** Spheroids in suspension are labelled with Click-iT EdU at time point 90 h and incubated for 6 h. After 96 h, spheroids were fixed and stained with Hoechst. Scale bars 60 µm. **(B)** Representative confocal microscopy images of phalloidin staining after 96 h show F-actin intensity expression (maximum projection (top); z-slice (bottom)). Scale bar 60 µm. **(C)** Schematic of the analysis method to quantify the ratio of F-actin distribution in the periphery and centre of the spheroid (top). An example output image of a spheroid after the analysis pipeline displaying mask creation and ROI extraction (bottom). **(D)** Quantification of the percentage of cells expressing EdU (n = 9 individual spheroids, N = 4 experiments, ****P< 0.0001, ***P< 0.0005, bars = mean and SD). Statistic test: ordinary one-way ANOVA, Šídák’s multiple comparisons test. **(E)** Quantification of the ratio of F-actin intensity in the spheroid area. The graph shows the distribution ratio related to the inner region of the spheroid area. Statistic test: unpaired t-test (n = 8 individual spheroids, N = 4 experiments, bars = mean and SD).

Next, we explored whether biochemical cues, including BMP4 signalling, influenced the proliferation of free-floating hiPSCs spheroids as the shape changes. This was investigated using an EdU proliferation assay on 3D spheroids in suspension. In E8 medium, cells were highly proliferative and exhibited homogenous EdU expression (Figures 3A, D). When spheroids were cultured in KSR BMP4 medium, cell proliferation was reduced and restricted to the tips of elongated spheroids (Figures 3A, D). In the intermediate conditions, the proliferation rate in E8 BMP4 was significantly decreased compared to E8, whereas spheroids cultured in KSR notably polarised toward the budding area (Figures 3A, D). These results indicate that biochemical cues in the medium regulate cell proliferation, suggesting that low cell proliferation is associated with BMP4 treatment. In contrast, KSR medium prompts proliferative cell polarisation, which induces morphogenesis and differentiation.

Having observed variation in the number of proliferative cells post-BMP4 treatment and under KSR, we next explored the F-actin network, which has been reported as an indicator of cellular tension (Clarke and Martin, 2021). Here, we postulate that cellular tension regulates spheroids’ morphology and can be inferred based on F-actin intensity and its orientation within spheroids (Figures 3B, C, E). Phalloidin staining revealed that spheroids cultured in E8 medium exhibited organised and tightly packed F-actin across the entire spheroid (Figure 3B). In KSR BMP4 conditions, we observed a disorganised and stretched F-actin network localised preferentially around the edges (Figure 3B). However, in spheroids cultured in E8 BMP4, F-actin accumulated in the core of the spheroids (Figure 3B). In KSR medium, we also observed a disorganised F-actin network stretching around the edges of spheroids (Figure 3B). Overall, these observations show that changes in morphology correlate with changes in cellular tension (F-actin organisation) under distinct medium conditions and may suggest a causal relationship between the two readouts. Following these indications, we quantified F-actin intensity distributions (Figure 3C). F-actin orientation changed upon treatment with different media. In KSR BMP4, elongated spheroids showed more F-actin expression in the periphery (Figure 3E). In E8 BMP4 and E8 conditions, the F-actin is distributed in the inner core (centrally). Altogether, these observations indicate that in differentiating conditions, KSR and KSR BMP4, cellular tension is associated with changes in morphogenesis.

### PEG-peptide hydrogel encapsulation disrupts SOX17 patterning

**FIGURE 4:**
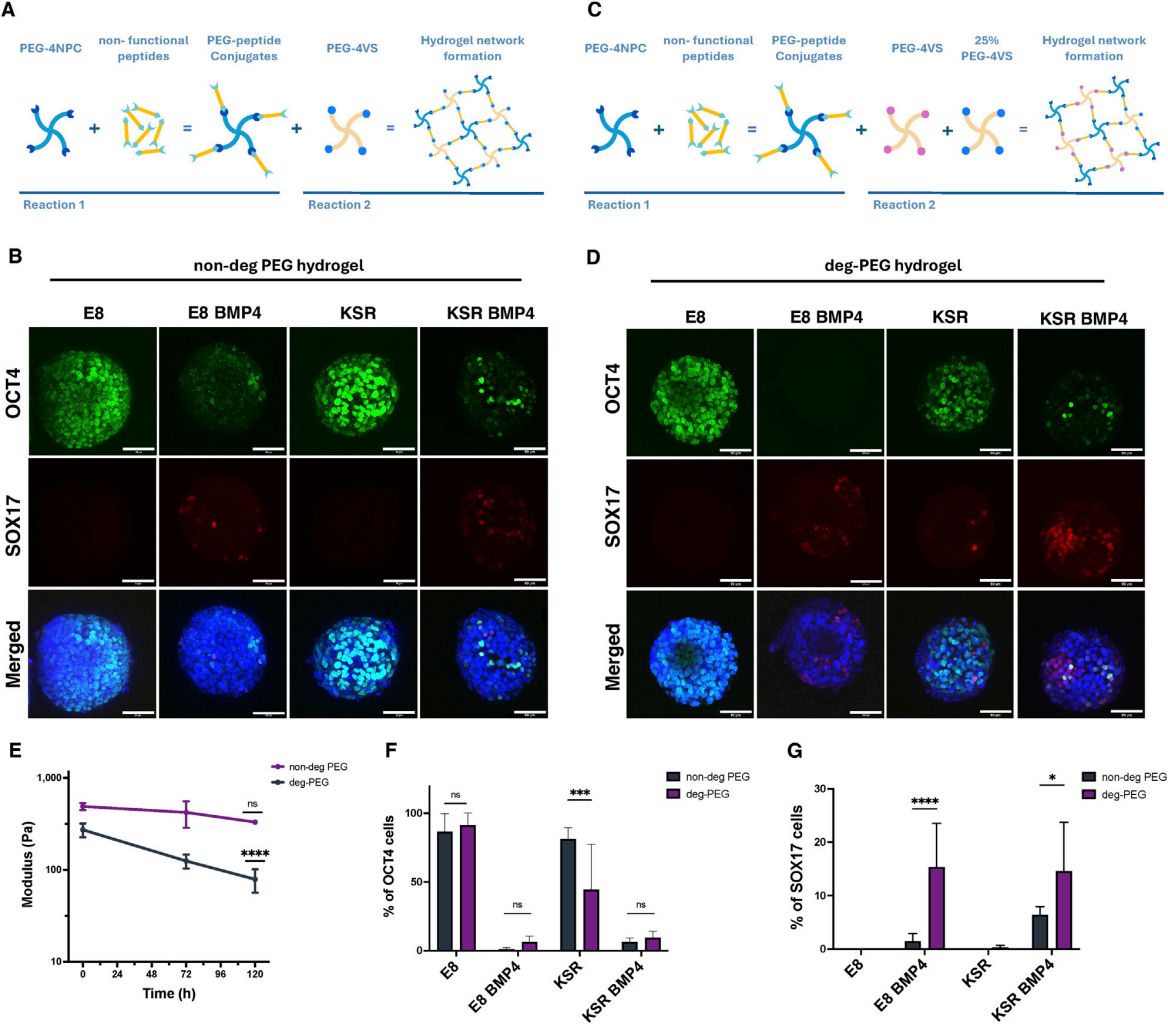
hiPSCs spheroids encapsulation in PEG hydrogels prevents morphological changes and affects the expression of SOX17. **(A)** Schematic diagram of PEG hydrogel formation based on two reactions. First, PEG-4NPC binds to a non-functional peptide (KDWERC) to form PEG-peptide conjugates. Conjugates are cross-linked with PEG-4VS to form the hydrogel network. **(B)** Confocal microscopy images of hiPSCs spheroids encapsulated in non-degPEG for 96 h: expression of OCT4 (pluripotency) and SOX17 (endodermal) markers. Scale bar 60 µm. **(C)** A schematic showing how hydrogels are modified to alter degradability by cross-linking the PEG-peptide conjugate with 75% PEG-4Acr and 25% PEG-4VS **(D)** Representative confocal microscopy images of OCT4 and SOX17 expression for hiPSCs spheroids after encapsulation in a deg-PEG for 96 h. Scale bar 60 *µ*m. **(E)** Quantification of hydrogel stiffness over time with rheological measurements. The graph shows that deg-PEG hydrogel softens significantly over time (n = 3 gels, ****P< 0.0001, bars = SD). **(F)** Quantification of the percentage of cells expressing OCT4 in non-deg PEG hydrogel compared to deg-PEG hydrogel cultured in E8, KSR BMP4, E8 BMP4 and KSR. Statistic test: 2-way Anova (n = 9 spheroids per condition, N = 3 experiments, ***p = 0.0005; bars = mean and SD). **(G)** Quantification of the percentage of SOX17 positive cells in non-deg PEG hydrogels showing a significant reduction in KSR BMP4 and E8 BMP4 compared to deg-PEG hydrogel, statistic 2-way Anova (n = 9 spheroids per condition, N = 3 experiments, ****P< 0.0001, *p = 0.01 bars = mean and SD).

We next aimed to investigate further whether there was a relationship between changes in shape and the patterning of germ layers in 3D hiPSCs spheroids. When spheroids were exposed to BMP4, we observed the emergence of SOX17-expressing cells, which coincided with axial elongation (Figures 1B, 2A). Therefore, we hypothesised that SOX17 expression was dependent on this shape change and that impeding elongation would impact the expression of SOX17. To test this, spheroids were physically confined using fully elastic PEG-based hydrogels. We harnessed our previously reported synthetic platform that could support the culture of hiPSC-derived intestinal organoids (Jowett et al., 2021; Norman et al., 2021). These PEG-based hydrogels are formed at low polymer concentrations using two sequential reactions. First, four-arm PEG nitrophenyl carbonate (PEG-4NPC) is conjugated with hetero-bifunctional peptides to form PEG-peptide conjugates. These conjugates are then cross-linked with four-arm PEG vinyl sulfone (PEG-4VS) to form the non-degradable hydrogel network (non-degPEG) (Figure 4A). We observed that confinement within non-degPEG prevented morphological changes in spheroids under differentiating conditions after BMP4 treatment (Figure 4B). Confined spheroids in E8 medium exhibited high levels of pluripotency marker OCT4, and no SOX17 expression was detected, similar to suspension conditions (Figures 4B, 2A). Interestingly, encapsulated spheroids in KSR BMP4 medium showed significantly reduced OCT4 and SOX17 expression (Figures 4B, 4F, G). Similarly, the E8 BMP4 condition showed a significant reduction in expression of OCT4 and very few SOX17 positive cells (Figures 4B, F, G). This was in contrast to the KSR medium condition in which spheroids maintained high expression of OCT4, but no SOX17 expression was detectable, which is comparable to the undifferentiated condition E8 (Figures 4B, 4F, G). These results indicate that when cultured within confining hydrogels, cells within hiPSC spheroids do not express SOX17 as they do in free-floating (suspension) differentiating conditions (Figures 2A, E). Overall, this suggests that embedding hiPSCs in a confining environment blocks morphogenesis, and despite the addition of BMP4, this was insufficient to prompt SOX17 expression.

### Modulating PEG-peptide degradability promotes SOX17 expression

The emergence of SOX17 lineage was dramatically reduced compared to free-floating culture when hiPSCs spheroids were cultured in confining hydrogels. We thus asked whether encapsulating spheroids in a hydrogel that was not confining would permit differentiation. We, therefore, modulated our design by swapping PEG-4VS with tetra-arm PEG molecules functionalised with acrylate groups (PEG-4Acr) to form a degradable hydrogel network (deg-PEG) (Figure 4C). PEG hydrogels cross-linked with acrylate have been reported to undergo controlled softening over time (Gjorevski et al., 2016). Using small amplitude oscillatory rheology, we confirmed that while standard PEG hydrogels cross-linked with PEG-4VS did not exhibit significant changes in stiffness over 5 days under standard culture conditions (G′ ∼ 488.3 Pa to 329.9 Pa) (Figure 4E), hydrogels formed by swapping 75% of the PEG-4VS with PEG-4Acr softened over 5 days and were significantly softer than day 0 controls (∼ 270 Pa to 78.7 Pa) (Figure 4E).

To determine whether encapsulation within deg-PEG hydrogels influences lineage differentiation, we stained for OCT4 and SOX17. Our findings show that in self-renewing conditions (E8 medium), encapsulated spheroids within deg-PEG maintained high expression of OCT4 and did not express SOX17 (Figure 4D). As expected, in the KSR BMP4 medium condition, we observed a significant reduction of OCT4 expression. We also identified an increase in SOX17 expression compared to non-degPEG (Figures 4B–D, F, G). Furthermore, when spheroids encapsulated in deg-PEG were cultured in E8 BMP4, OCT4 was expressed in very few cells; however, we did observe a significant increase in SOX17 expression compared to non-degPEG cultures (Figures 4B–D, F, G). Spheroids grown in the KSR medium showed reduced expression of OCT4 in deg-PEG compared to non-degPEG conditions (Figures 4D, F), and SOX17 expression remained low (Figures 4D, G). Altogether, these findings demonstrate that deg-PEG conditions partially restored SOX17 expression in both the KSR BMP4 and E8 BMP4 conditions.

## Discussion

The connection between form and function is a long sought-after problem in biology and beyond (Hennig, 2020). In early development, the specification of three germ layers is controlled by morphogen signalling and physical forces between cells and the local tissue environment (Muncie et al., 2020; Seitz et al., 2024). The physical properties of the native tissue vary (0.2–1 kPa) during developmental stages *in vivo,* which regulate the self-renewing and differentiation process during early development (Muncie et al., 2020; Rasmussen et al., 2016; Fujii et al., 2021; Vining and Mooney, 2017; Abbas et al., 2019). Nevertheless, the interconnection of patterning, cell fate and morphogenesis during human development remains difficult to untangle. Spheroids formed from hPSCs present an intriguing model of this process as they have been shown to undergo elongation, and their cells differentiate to form the three germ layers resembling the early stages of development (Moris et al., 2020; Arias et al., 2022). Here, we compare self-renewing versus differentiation conditions in suspension cultures of 3D hiPSCs spheroids, which give rise to morphological changes and the emergence of lineage specification. Our results robustly demonstrate that biochemical cues trigger morphological changes forming spherical shapes in E8, and axial elongation in KSR BMP4. However, in their control conditions, E8 BMP4, spheroids maintained a spherical shape while forming small buddings, and when spheroids were cultured in KSR, intermediate shapes formed, undergoing spontaneous budding.

Various culture conditions, including feeder and feeder-free conditions (Nutristem, E6 or serum replacement KSR) have been widely exploited to maintain hPSCs cultures (Muncie et al., 2020; Deglincerti et al., 2016; Prakash Bangalore et al., 2017). Moreover, several studies have investigated the role of Wnt signalling in inducing tri-lineage formation using BMP4 in 2D micropattern systems (Muncie et al., 2020; Warmflash et al., 2014; Tewary et al., 2017; Vickers et al., 2021). However, in 3D models, hESCs treated with BMP4 in E6 medium did not show the ability to elongate and differentiate, whereas pre-treating the cells with the Chiron -Wnt agonist initiated axial elongation and germ layers formation (Moris et al., 2020; Baillie-Benson et al., 2020; Chhabra et al., 2019). We have demonstrated that 3D hiPSCs treated with BMP4 under KSR medium conditions robustly break symmetry, elongate and generate tri-germ layers. Cells in the KSR BMP4 medium spontaneously elongate mirroring A-P elongation, and consistently present polarisation of SOX17 expression (endoderm) at the tip of the extended elongation. They also express BRA (mesoderm) and SOX2 (ectoderm). On the other hand, spheroids cultured in E8 medium express high level of OCT4, compatible with the cell population retaining pluripotency (Amita et al., 2013). Moreover, we found that OCT4 is significantly reduced in the differentiation condition in which lineage markers are expressed. Analysis of NANOG might provide further evidence for retention/loss of the pluripotent state (Amita et al., 2013; Mathew et al., 2012). This verifies that our media conditions can effectively maintain pluripotency or drive differentiation. Notably, spheroids cultured in E8+BMP4 failed to elongate, and SOX17 expression occurred randomly. This effect has been reported previously in both human and mouse pluripotent stem cells when exposed to BMP4 (Amita et al., 2013; Mathew et al., 2012; Mfopou et al., 2014). Altogether, this suggests that adding BMP4 to the KSR medium in our model is sufficient to induce morphogenesis and differentiation toward the three germ layers, consistent with published 2D micropattern models (Warmflash et al., 2014; Tewary et al., 2017; Vickers et al., 2021).

Following observations suggesting that germ layer differentiation was associated with shape changes in free-floating culture, we aimed to impede this process using PEG hydrogels. We found that physical confinement prevents morphological changes and dramatically reduces the expression of SOX17 in both KSR BMP4 and E8 BMP4, with a loss of BRA expression in KSR BMP4. We then sought to identify if modulating hydrogel degradability could promote morphogenesis and differentiation. Deg-PEG hydrogels did not promote elongation and yet stimulate SOX17 but not BRA expression in KSR BMP4 and SOX17 in E8 BMP4 conditions. The formation of BRA positive cells (mesoderm) occurs early in gastrulation when the cells ingress and establish axial elongation (Pour et al., 2022; Schüle et al., 2023). This suggests that cellular movement to develop axial elongation is required to obtain proper germ layers specification. A time course experiment to validate the lineage markers expression via immunostaining at early and late time points would provide additional readouts on dynamics (Moris et al., 2020; Fujii et al., 2021). However, further investigation is required to quantify these markers’ expression and explore cell type identities and lineages present in this model, possibly via transcriptomics analysis (Moris et al., 2020; Tyser et al., 2021; Han et al., 2018). Nevertheless, our approach allowed us to observe the inter-correlation of morphological changes and the patterning of germ layers in 3D hiPSCs spheroids.

Cells generate tension via the cytoskeleton that drives changes in tissue morphology (Grolleman et al., 2023; Kim et al., 2018). In the E8 medium condition, F-actin was distributed centrally, suggesting that tension was homogeneous and perhaps pushed the sphere to expand. F-actin was distributed around the periphery in both KSR and KSR BMP4 conditions, indicating higher tension around the edges, especially in the elongated area. These observations suggest that in such differentiation conditions (KSR and KSR BMP4), cellular tension might contribute to morphogenesis and is in keeping with reports that the polymerisation of the F-actin network toward the plasma membrane generates pushing forces resulting in shape changes (Grolleman et al., 2023; Clarke and Martin, 2021).

This work focussed on investigating how biochemical cues and physical confinement separately influence morphogenesis and germ layers specification in 3D hiPSCs-derived models of gastrulation. These outcomes open opportunities for further investigation to characterise cell lineages expressed upon BMP4 treatment, and to explore physical forces generated by cell-cell adhesion or actomyosin contractility. Indeed, we previously showed that these models are suitable for highlighting the relationships between genetic variations and germ layer differentiation for selected hiPSCs lines (Vickers et al., 2021), establishing a valuable 3D model to test other cell lines further. Moreover, by modifying hydrogel mechanical properties, we explored how physical confinement impacted lineage differentiation and morphogenesis during early embryogenesis. Thus, 3D models of hiPSCs spheroids combined with tunable PEG-peptide hydrogels can be used to understand the effect of the microenvironment on mechanosignalling, and polarisation during early development.

## Methods

### hiPSCs culture

hiPSCs line (Hoik_1) were selected from HipSci biobank (www.hipsci.org). As previously described (Kilpinen et al., 2017), hiPSCs were derived from skin fibroblast and reprogrammed using Sendai virus vectors (CytoTune) expressing the four factors OCT4, SOX2, MYC and KLF4. Briefly, iPSCs were cultured on a previously coated 6-well plate (Thermo Fisher Scientific) with 10 μ g/mL vitronectin (Stem cell Technologies). Cells were cultivated in feeder-free medium using Essential 8 (E8) medium with 2% E8 supplement (50x) (Thermo Fisher Scientific), and 1% Penicillin Streptomycin 5,000 U/mL (Gibco) incubated at 37°C, 5% CO_2_. Cells were used at passages 28–31 and were not cultured for more than 6 passages for experiments.

### Spheroids derivation from hiPSCs

When colonies reached 70% confluency, typically after 3 days, cells were washed with Hank’s Balanced Salt solution (HBSS) (Gibco). Cells were incubated in Accutase (Bio Legend) for 3 min at 37° C, 5% CO_2_, and resuspended in E8 medium (Thermo Fisher Scientific) and 10 μM Y-27632 Rho-kinase inhibitor (ROCKi) (ENZO Life Sciences). Prior to cell seeding, low attachment 96-well V-bottom plates (Thermo Fisher Scientific) were coated with 5% (w/v) Pluronic solution (Sigma) as previously described (Alsehli et al., 2021). Plates were centrifuged at 500 *g* for 5 min and incubated at room temperature (RT) for 30 min, followed by washing with Phosphate Buffer Saline (PBS) (Gibco). The chosen cell seeding density was optimised for size and morphology consistency. Single cells were seeded at a density of 750 cells/well and cultured in E8 medium (Thermo Fisher Scientific) as described previously and 10 μM ROCKi (ENZO Life Sciences) to prevent cell apoptosis and enhance cell viability. hiPSCs were incubated for 48 h with a sufficient medium at 37° C, 5% CO_2_ to form spheroids (Figure 1A).

### Germ layers differentiation induction of 3D hiPSCs

The spheroids formation protocol was modified and adapted from previous studies (Ungrin et al., 2008; van den Brink et al., 2014). After 2 days in culture, the spheroids were formed, and medium was replaced either to self-renewing (1), differentiation condition (2) or their controls (3,4) at 37° C, 5% CO_2_ for 96 h, as follows; (1) E8 medium supplemented with 2% E8 supplement (50x) (Thermo Fisher Scientific), 1% Penicillin Streptomycin 5,000 U/mL (Gibco), and 10 μM ROCKi (ENZO Life Sciences); (2) Knockout serum medium (KSR) consisting of Advance DMEM/F-12 medium, supplemented with 20% Knockout serum replacement, 1% L-Glutamine, 1% Penicillin Streptomycin 5000 U/mL (all Gibco), 0.1 mM β-mercaptoethanol (Sigma), 10 ng/mL basic fibroblast growth factor (bFGF), 50 ng/mL BMP4 (Invitrogen), and 10 μM ROCKi (ENZO Life Sciences). (3) E8 medium composition as in (1) supplemented with 50 ng/mL BMP4; and (4) KSR medium as described in (2) with no addition of 50 ng/mL BMP4 (see Figure 1A).

### Hydrogel fabrication

Hydrogels were synthesised as previously described (Jowett et al., 2021; Lust et al., 2021) using 4-arm PEG-peptide conjugates and 4-arm PEG-VS 20 kDa (non-degradable) or a mixture of 25% 4-arm PEG-VS and 75% 4-arm PEG-4Acr 20 kDa (degradable) (see Figures 4A, C). Briefly, peptide conjugates were synthesised with Ac-KDW-ERC-NH2, >98% purity (custom synthesis Peptide Protein Research, Ltd. (United Kingdom)) and 4-arm 10 kDa PEG-NPC (JenKem Technology, United States). The N-terminal primary amine (lysine) of Ac-KDW-ERC-NH_2_ was reacted with PEG- NPC to form conjugates. Purified conjugates were cross-linked with either PEG-4VS or PEG-4Acr to form the hydrogel network through a Michael addition. Here, the reaction between peptide conjugate and cross-linkers (PEG-4VS and PEG-4Acr) was performed in a stoichiometric ratio 1:1 in 30 mM HEPES buffer at pH 8 (Sigma) diluted in 1x HBSS (Gibco). Hydrogels were allowed to form for 30–45 min. The solid content of the polymer was 2.5% (w/v) for both hydrogel conditions.

### Rheological measurements of hydrogels

Hydrogel storage modulus G′ was measured on a strain-controlled ARES from TA Instruments using a parallel plate geometry (8 mm plate). 50 μL hydrogels were prepared in 8 mm glass rings and placed onto the rheometer plate, and measurements were carried out at 37 °C while sealed with paraffin oil to prevent evaporation. A frequency sweep was recorded, measuring *G*′ as a function of shear frequency in the range 100–0.1 rad s^−1^ at a fixed strain of 1%. (Orchestrator software, version 7.2.0.2).

### hiPSCs spheroids encapsulation in PEG-peptide hydrogel

hiPSCs spheroids were obtained 48 h post seeding in 96-well V-bottom plate as previously described. The spheroid in each well was washed once with 30 mM HEPES buffer (pH 8.0), then resuspended in 30 mM HEPES buffer (pH 8.0) (Sigma). Spheroids were encapsulated within 10 μL of hydrogel, formed as described above in μ-slides angiogenesis glass bottom (ibidi) by placing the mixture in each well and incubating for 30–45 min at 37°C, 5% CO_2_. Medium containing 10 μM ROCKi (ENZO Life Sciences) was added, and cells were cultured for 96 h at 37°C, 5% CO_2_ replenish medium daily without BMP4.

### Live imaging

Time-lapse imaging was performed at 37°C, 5% CO_2_ using JuLI™ Stage Real-Time Cell History Recorder (NanoEnTek). hiPSCs-derived spheroids in 96-well V-bottom plate in suspension and encapsulated in PEG-peptide hydrogels were imaged every hour for 4 days (96 h). Brightfield time-lapse images were acquired by ×10 objective, using the following set-up: exposure time = 65 ms, brightness = 18, and focus at ∼8,851 to capture spheroids morphological changes over time. The setup could vary slightly between experiments, which require adjustment for focus and brightness.

### Immunofluorescence staining

Spheroids in suspension were fixed using 4% paraformaldehyde (PFA) (Sigma) incubated at room temperature (RT) for 45 min followed by three washes with PBS 5 min/wash. Cells were permeabilised and blocked with 0.3% Triton X100 (Sigma) in PBS, and 3% bovine serum albumin (BSA) (Sigma) for 1 h at RT on a shaker. Primary antibodies used were Rabbit anti-OCT4, Goat anti-SOX17, Goat anti-BRA, Goat anti-SOX2 (R&D); Mouse anti-YAP1 (Santa Cruz) were antibodies diluted in the blocking buffer, and spheroids were incubated in primary antibodies overnight in the dark at 4°C. Following three washes with 0.1% TritonX100 in PBS 5 min/wash, cells were incubated in secondary antibodies anti-rabbit Alexa Fluor 488, anti-goat Alexa Fluor 633, and Alexa Fluor 555 Phalloidin (Thermo Fisher Scientific) and DAPI, for 2 h RT on a shaker. Finally, spheroids were washed three times with 0.1% TritonX100 in PBS for 10 min/wash.

Encapsulated spheroids in PEG-4VS and 75% PEG-4Acr were fixed with 4% PFA incubated at RT for 45 min. Then, cells were washed three times with PBS for 10 min/wash, then permeabilised using 0.3% Triton X100 in PBS for 1 h. Cells were incubated in a blocking buffer consisting of 0.1% Triton X100 and 3% BSA overnight at 4°C. Primary antibodies: Rabbit anti-OCT4 (abcam); Goat anti-SOX17, and Goat anti-BRA (R&D); were diluted at 1:50 in the blocking buffer; cells were stained and incubated for 36 h in the dark at 4°C. Following five extensive washes using 0.1% Triton X100 in PBS for 10 min/wash, cells were stained with secondary antibodies at 1:50 anti-rabbit Alexa Fluor 488 and anti-goat Alexa Fluor 633 (Thermo Fisher Scientific) and DAPI overnight in the dark at 4°C. Then, cells were washed five times with 0.1% TritonX100 in PBS for 10 min/wash.

### Cell proliferation assay

Spheroids in suspension at day 4 were labelled with 4 μM EdU and incubated for 6 h using a Click-iT EdU Alexa fluor 488 imaging kit (Thermo Fisher Scientific). Spheroids were fixed with 4% PFA, incubated at RT for 45 min and washed three times with PBS. Cells were then blocked and permeabilised using 3% BSA and 0.3% TritonX-100 in PBS for 1 h at RT, followed by washing three times with 3% BSA. A click-iT reaction buffer was prepared (following the manufacturer’s instructions) at the desired volume. Cells were incubated in the reaction buffer for 30 min at RT, followed by washing once with 3% BSA. Nuclear staining, Hoechst 33342 (1:1000, Invitrogen) was added to the cells and incubated for 30 min at RT protected from light. Then, cells were washed twice with PBS for 5 min/wash.

### Imaging

Spheroids in suspension culture were transferred into a 96-well plate with a flat bottom, μclear black for imaging (Greiner). A Z-stack images of the selected markers and cell viability were acquired using Leica TCS SP8 confocal microscope with a ×40 oil objective. Encapsulated spheroids in μ-slides angiogenesis glass bottom (ibidi) were imaged for markers expression and live/dead assay. Embedded spheroids were imaged with a ×63 oil objective. Image processing was performed and visualised using ImageJ version 2.0.0 (Fiji) and OMERO—insight Version 5.5.9.

### Image analysis

Image analysis was performed using Imaris software version 9.9. A surface mask was created on the DAPI channel to measure sphericity after changes in morphology. This provides readouts for changes in shape via sphericity and deformation values (values range closer to 1 corresponds to spherical structure, while values below 0.7 indicate shape deformation (Szmańda and Witkowski, 2021).

Nuclei were quantified using spot-counting methods, pluripotency, and cell proliferation markers to determine the germ layer expression intensity. The percentage of positive expression was determined from the total number of cells in the DAPI channel for the respective spheroid. Colocalisation of OCT4 and SOX17 was calculated based on expression thresholds for co-expressed markers, and then spot detection was used to quantify the number of cells.

To analyse the F-actin distribution around the spheroids, a pipeline was created in Icy software, an open community platform for bioimage informatics (de Chaumont et al., 2012) (Figure 3C). Pre-processing of the images included conversion into maximum intensity projections (MIP) and a Gaussian blur to improve the segmentation quality of the whole spheroid area based on the DAPI channel and the F-actin network based on the F-actin channel. First, the two channels (DAPI and F-actin) were extracted separately. The DAPI channel was used for segmentation to detect the whole spheroid area using HK-means thresholding, creating a mask of the entire spheroid. Here, in the HKmeans method, gaussian pre-filter (value = 3) was used to remove noise, and the minimum object size was selected at 10,000 pixels to generate a region of interest (ROIs) that represents the whole spheroid area. As DAPI provides consistent and reliable staining, using this channel for mask creation improves the quality and reliability of segmentation and removes any bias due to changes in intensity in the F-actin channel. Next, A “fill-holes in ROI” step was included to create a single mask that evenly covered the spheroid’s whole area. Therefore, we determine our ROIs by dividing the entire area mask into an inner ring area (to measure F-actin within the centre of the spheroid) and an outer ring area (measuring F-actin within the periphery). To create these ROIs, for each image, the whole area mask was reduced by a scale percentage of 25 along both the *X* and *Y*-axis to reduce the ROI to the central region in the inner core. Next, the inner ROI was subtracted from the whole area mask to obtain the outer ring region. Reduction of each mask by a set scale percentage of 25 was chosen to remove bias and maintain as even as possible volume within each ROI. After identifying the ROI, the F-actin channel was used as input to extract the average intensity within each ROI area. The pipeline was run in an automated manner to remove bias (see details pipeline in Supplementary Image S3).

### Statistical analysis

Data from the live images pipeline and Imaris software were compiled into Microsoft Office 365 Excel 16.62. Data were exported to GraphPad Prism version 9.3.1., Results are represented as means with standard deviation (SD). Statistical analysis was performed via ordinary one-way ANOVA test for multiple comparisons (Šídák test) to analyse markers expression (OCT4, SOX17, SOX2, and BRA) between medium conditions. An unpaired t-test was used to analyse F-actin staining intensity and hydrogel shear modulus measurements.

## Data Availability

The original contributions presented in the study are included in the article/Supplementary Material, further inquiries can be directed to the corresponding authors.

## Supplementary material

SUPPLEMENTARY IMAGE S1 Tracking axial elongation via live imaging. Spheroids cultured in KSR BMP4 medium at different time points show changes in morphology; at 36 h, they start to elongate. Tracking the direction of spheroid elongation allows us to identify the assumed anterior-posterior domain.

SUPPLEMENTARY IMAGE S2 YAP localisation in hiPSC. Representative confocal microscopy images for hiPSCs spheroids cultured in E8 vs. KSR BMP4 medium for 96 h. Immunostaining of YAP localisation showed cytoplasmic YAP in E8 medium (top), in KSR BMP4 (bottom) condition YAP localised to the nucleus as indicated in the magnification images (z-slice). Scale bar 60 µm.

SUPPLEMENTARY IMAGE S3 F-actin quantification method. A diagram of the Step-by-step pipeline adapted from Icy software.
