# Supplementary figures and images for: Morphogen-driven differentiation is precluded by physical confinement in human iPSCs spheroids
Source: Front Bioeng Biotechnol. 2024 Nov 11;12:1467412. doi: 10.3389/fbioe.2024.1467412 (PMC11586224; doi:10.3389/fbioe.2024.1467412)

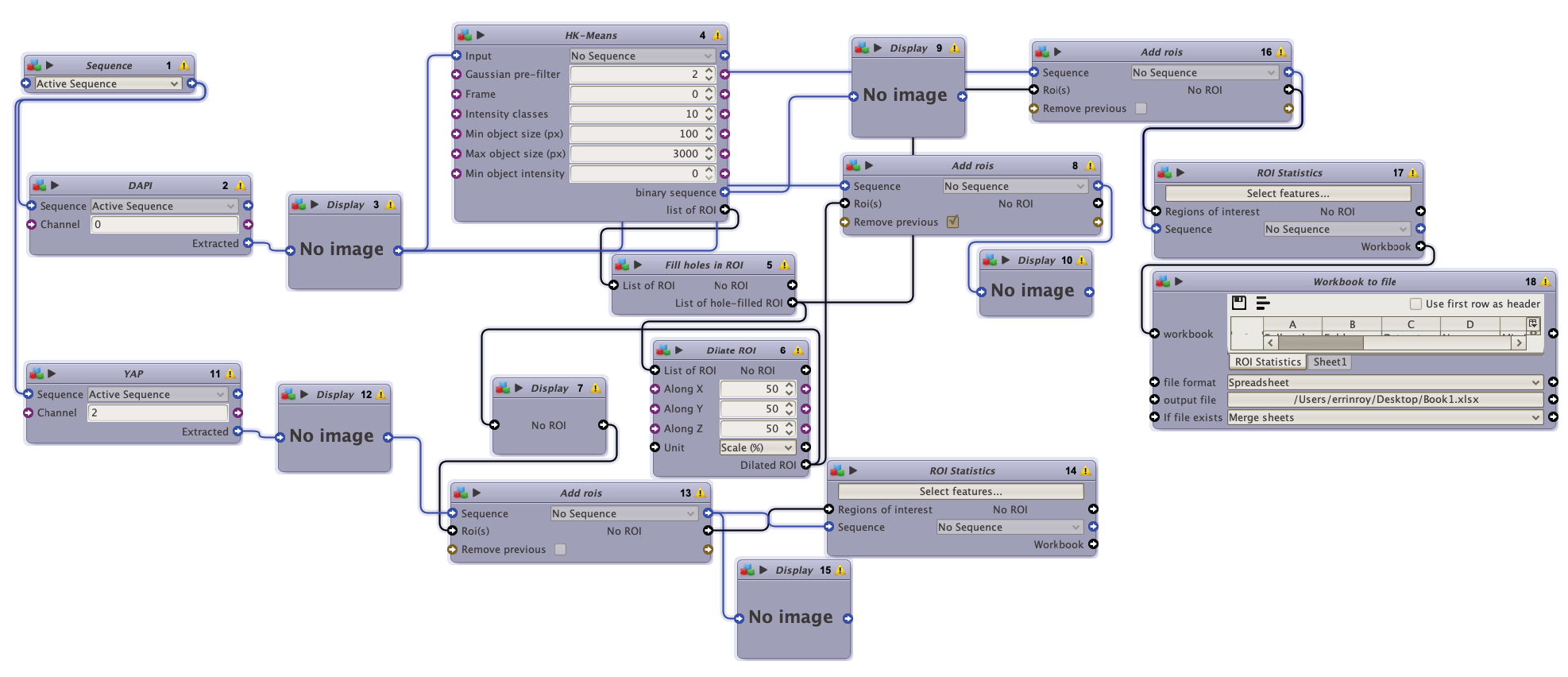

Supplement: Supplementary file 1 [file Image3.TIFF]

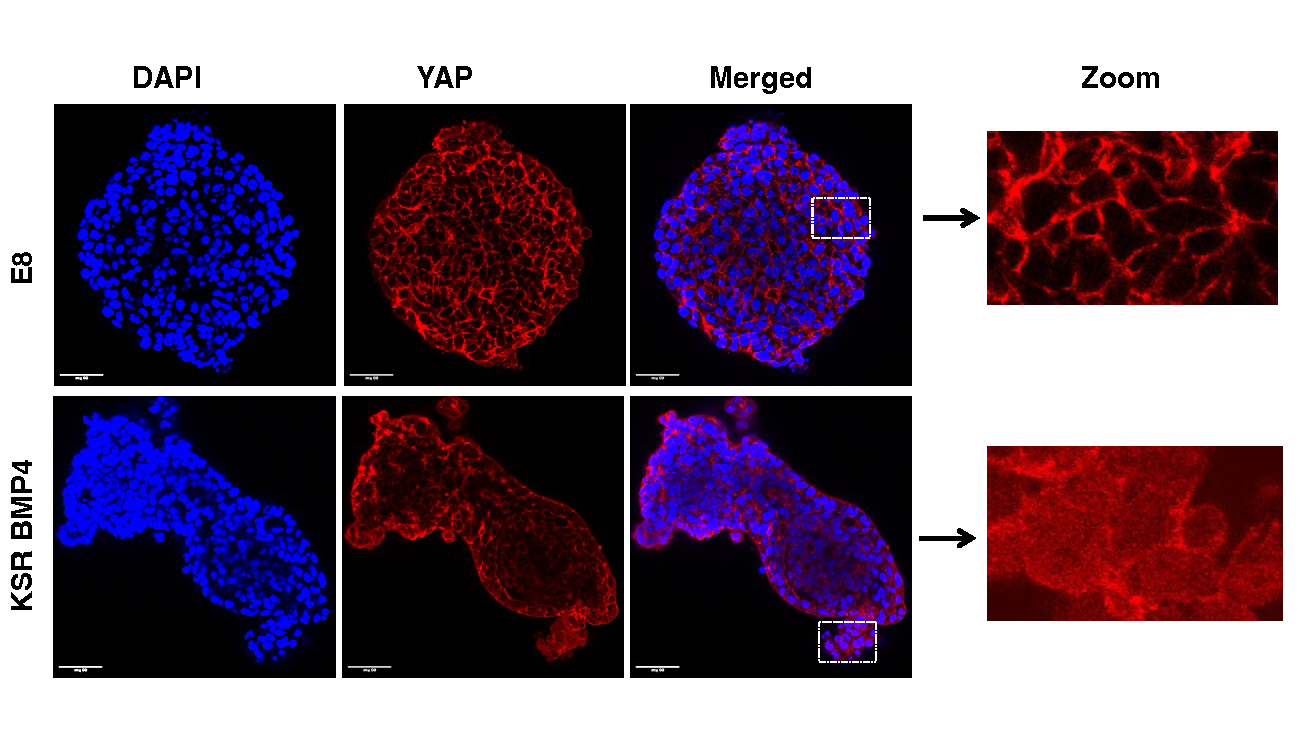

Supplement: Supplementary file 6 [file Image2.TIFF]
